# Buried Bumper Syndrome With Full Gastric Wall Penetration Managed With Delayed Replacement: A Conservative Approach

**DOI:** 10.7759/cureus.92137

**Published:** 2025-09-12

**Authors:** Hao The K Nguyen, Benjamin Pfeiffer, Toma Evanoff, Jaren Yatsu, Michelle Kem Hor

**Affiliations:** 1 College of Osteopathic Medicine, Rocky Vista University, Parker, USA; 2 Gastroenterology, UCHealth Memorial Central Hospital, Colorado Springs, USA

**Keywords:** buried bumper syndrome, endoscopy, gastric perforation, peg replacement, rupture

## Abstract

Buried bumper syndrome is an uncommon but serious complication of gastrostomy tube placement, in which the internal bumper migrates into the gastric wall or beyond. Complications include bleeding, perforation, peritonitis, and death. We present a case of a 21-year-old man with Batten disease who presented with abdominal pain, swelling, and leakage from his gastrostomy tube. Imaging revealed that the balloon had advanced through the gastric wall, and an endoscopy was performed. Endoscopic examination revealed buried bumper syndrome with full gastric wall penetration. The tube was removed, and replacement was delayed for eight days to allow mucosal healing, with proton pump inhibitors used during this time. A new tube was successfully replaced. This case highlights an important learning point: delayed replacement can be a viable strategy in avoiding surgery in buried bumper syndrome when endoscopic placement is not otherwise feasible.

## Introduction

Buried bumper syndrome (BBS) is an uncommon complication in which the internal bumper of a percutaneous endoscopic gastrostomy (PEG) tube migrates from the gastric lumen into the gastric wall or beyond [1]. This typically occurs due to excessive compressive force between the internal and external fixations of the tube. BBS can lead to serious complications, including gastrointestinal bleeding, perforation, abscess formation, peritonitis, and even death [2]. The majority of BBS cases are managed with endoscopic techniques, while surgical intervention is reserved for more severe presentations [3]. A retrospective multicenter study published in 2024 analyzed 160 BBS cases and their management. The authors found that 143 of these cases were managed endoscopically, 9 without removal, and 8 PEG tubes were surgically replaced [4]. However, none of these cases featured a delayed endoscopic replacement strategy such as in our patient. We report a case involving a 21-year-old with BBS featuring complete gastric wall penetration managed conservatively, with replacement after mucosal healing.

## Case presentation

**Figure 1:**
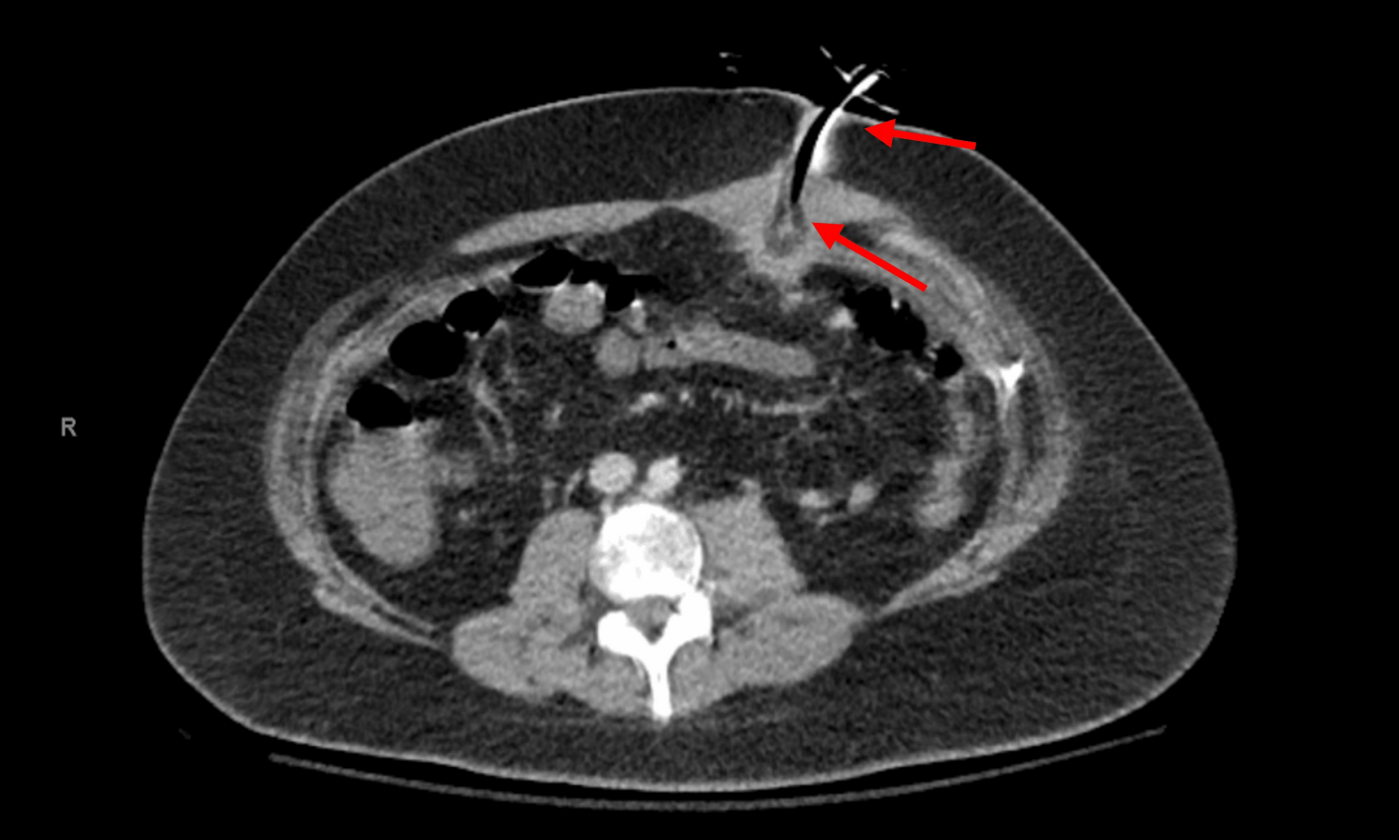
CT scan showing increased density around the percutaneous endoscopic gastrostomy (PEG) tube, with the balloon possibly located outside the gastric lumen.

A 21-year-old man with Batten disease and seizure disorder was brought to the emergency department with worsening pain, swelling, and leakage around his PEG tube for several weeks. The PEG tube had been placed seven months earlier. The patient was nonverbal and bedbound. Vital signs, white blood cell count, hemoglobin, and hematocrit were unremarkable. A CT scan of the abdomen and pelvis suggested that the PEG tube was outside the gastric lumen (Figure 1).

**Figure 2:**
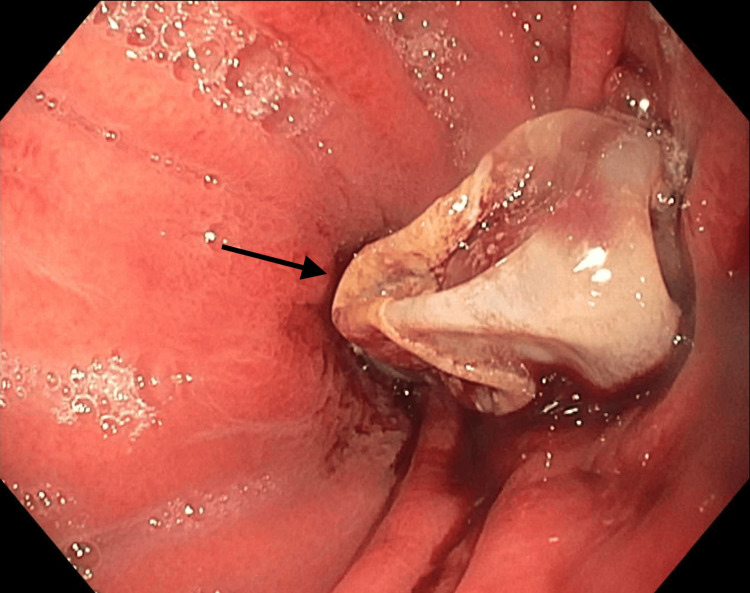
Gastric body with balloon advanced in the stomach after decompression.

**Figure 3:**
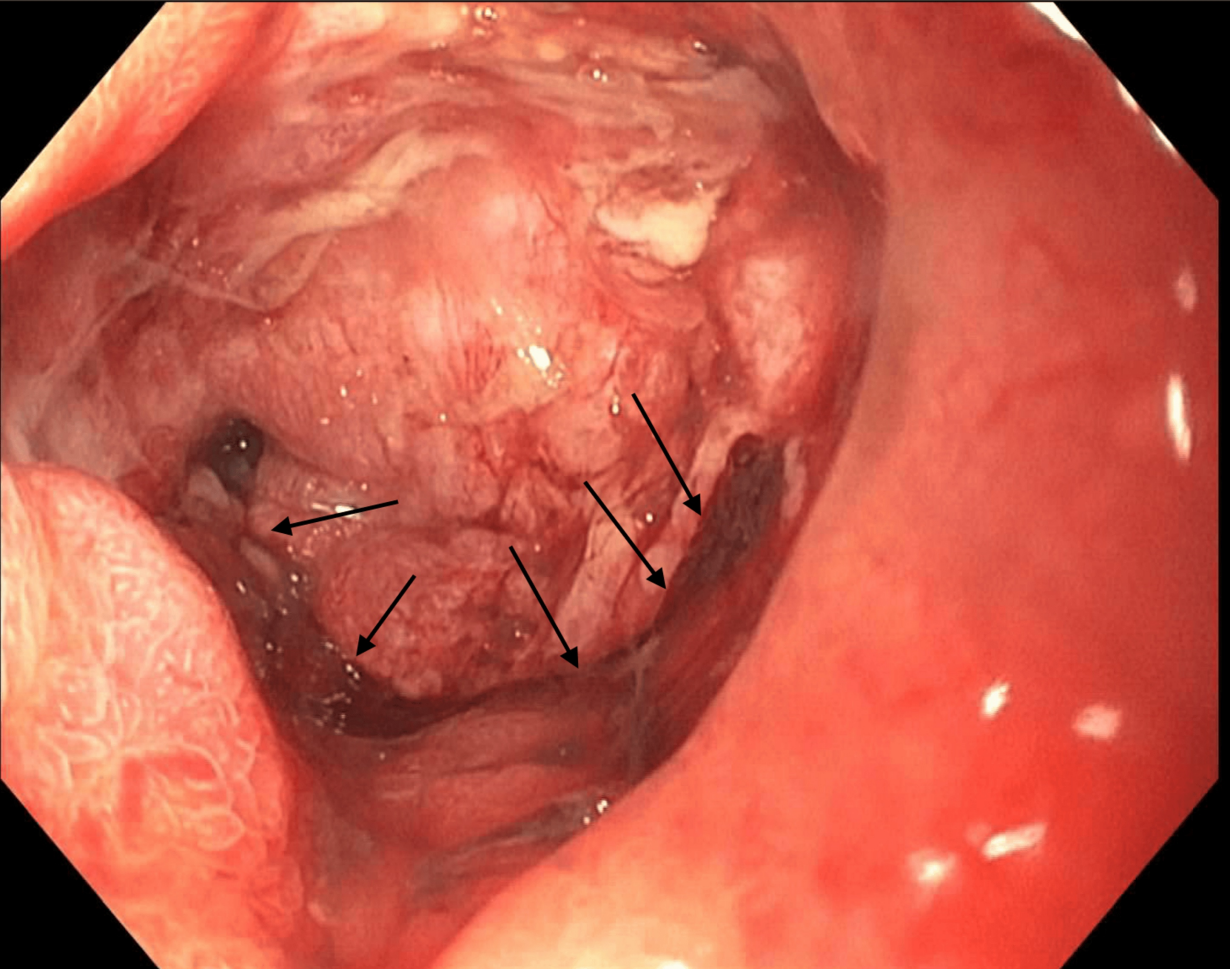
Gastric body with a deep compression ulcer at the percutaneous endoscopic gastrostomy (PEG) site

On endoscopic examination, the tube was found on the anterior wall of the gastric body. The balloon had deeply embedded into the gastric wall (Figure 2). Upon removal, complete penetration through the gastric wall into fibrosed peritoneum was observed (Figure 3). A new feeding tube was not placed because of the presence of a walled-off deep compression ulcer.

The patient was started on total parenteral nutrition (TPN), and topical antibiotics were applied at the PEG site. A proton pump inhibitor (PPI) was initiated to aid in ulcer healing. Given his poor surgical candidacy, the plan was to allow one week for healing before re-evaluation.

**Figure 4:**
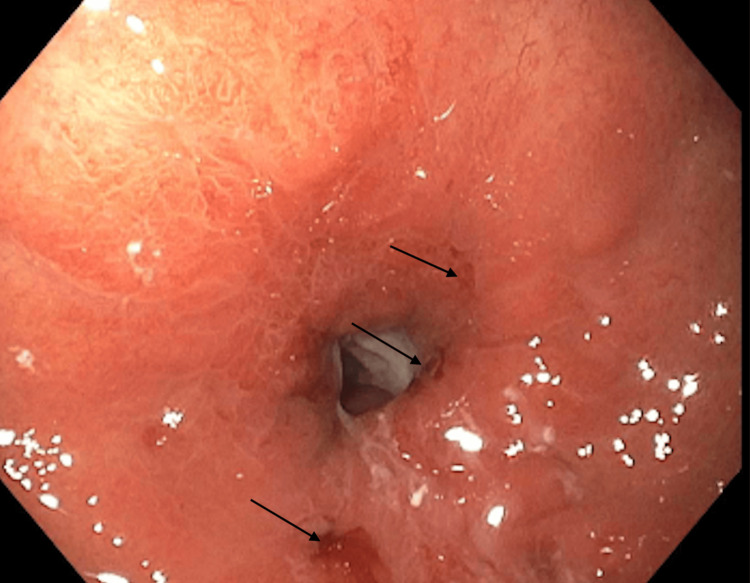
Healed gastric mucosa following an eight-day delay in percutaneous endoscopic gastrostomy (PEG) tube replacement, with minor lesions remaining.

Eight days later, repeat endoscopy showed a well-healed gastric body with a remaining 8-mm nonbleeding ulcer (Figure 4). A guidewire was placed using an endoscope and snare apparatus. A skin incision was made at the trocar needle site, and the gastrostomy tube was advanced through the oral cavity into the stomach. The trocar needle was removed, and the tube was pulled through the abdominal wall. The external bumper was secured, and the guidewire was removed. Follow-up endoscopy confirmed correct tube positioning. The skin marking at the external bumper was noted to be 5 cm. Tension and abdominal wall compression were verified, ensuring the bumper was loosely resting against the skin. The tube was capped, and the site was cleaned and dressed.

The patient tolerated the procedure well, showing no signs of distress. The feeding tube was monitored for three additional days without complications before discharge.

## Discussion

Buried bumper syndrome is a serious complication of PEG tube placement, with an incidence ranging from 0.3% to 2.4% [2]. Endoscopic intervention is the most commonly used treatment, particularly the push/pull technique (PPT) and non-papillotome technique (NPT) [5]. Failed primary treatment may necessitate surgical intervention or, in some cases, management without tube removal. In severe cases, complications such as wound infection, peritonitis, necrotizing fasciitis, and death have been reported [6,7].

A retrospective analysis of 136 patients with BBS showed that 70 had a replacement tube placed through the existing tract, 28 had a new puncture site, and 38 retained their original PEG tube [5]. However, literature is sparse regarding cases involving full PEG removal and delayed replacement, as seen in our patient.

A retrospective review of 641 PEG placements revealed that 3.9% of patients developed major complications, with 15.8% of those due to BBS [8]. Mortality rates following PEG tube placement range from 5% to 15%, depending heavily on indication and comorbidities [9,10]. Although the incidence of BBS is known, no studies directly quantify mortality specific to this complication. Its rarity and potential underreporting may contribute to limited data.

## Conclusions

Due to the iatrogenic nature and potential severity of BBS, prevention is critical. This includes proper external bumper positioning, periodic loosening and rotation of the PEG tube, and attentive wound care. Our report of this 21-year-old man with Batten disease highlights the importance of educating family members and healthcare professionals on the signs of PEG tube complications such as BBS. It also demonstrates that a nonsurgical, delayed replacement strategy may be a viable approach in carefully selected patients who are poor or moderate surgical candidates.

## References

[1] Menni A, Tzikos G, Chatziantoniou G (2023). Buried bumper syndrome: a critical analysis of endoscopic release techniques. World J Gastrointest Endosc.

[2] Cyrany J, Rejchrt S, Kopacova M, Bures J (2016). Buried bumper syndrome: a complication of percutaneous endoscopic gastrostomy. World J Gastroenterol.

[3] Dixon P, Kowdley GC, Cunningham SC (2016). The role of surgery in the treatment of endoscopic complications. Best Pract Res Clin Gastroenterol.

[4] Steinbrück I, Pohl J, Friesicke M (2025). Treatment of the buried bumper syndrome: a retrospective multicenter study with inclusion of 160 cases. J Clin Gastroenterol.

[5] Anagnostopoulos GK, Kostopoulos P, Arvanitidis DM (2003). Buried bumper syndrome with a fatal outcome, presenting early as gastrointestinal bleeding after percutaneous endoscopic gastrostomy placement. J Postgrad Med.

[6] Biswas S, Dontukurthy S, Rosenzweig MG, Kothuru R, Abrol S (2014). Buried bumper syndrome revisited: a rare but potentially fatal complication of PEG tube placement. Case Rep Crit Care.

[7] Jafari A, Weismüller TJ, Tonguc T, Kalff JC, Manekeller S (2016). Complications after percutaneous endoscopic gastrostomy tube placement - a retrospective analysis (Article in German). Zentralbl Chir.

[8] Anderloni A, Di Leo M, Barzaghi F (2019). Complications and early mortality in percutaneous endoscopic gastrostomy placement in lombardy: a multicenter prospective cohort study. Dig Liver Dis.

[9] Stenberg K, Eriksson A, Odensten C, Darehed D (2022). Mortality and complications after percutaneous endoscopic gastrostomy: a retrospective multicentre study. BMC Gastroenterol.

[10] Clarke E, Pitts N, Latchford A, Lewis S (2017). A large prospective audit of morbidity and mortality associated with feeding gastrostomies in the community. Clin Nutr.

